# Hospitals and the Duty of Business Firms

**Published:** 1903-07-25

**Authors:** 


### HOSPITALS AND THE DUTY OF BUSINESS FIRMS.

### A LIMITED COMPANY MAKES A GRANT TO A HOSPITAL.

In the course of his speech to the shareholders in Lovell and Christmas, Limited, at the annual meeting on the 15th inst., Mr. J. C. Lovell, the Chairman, after dealing with the report and accounts, said:—Now I come to another point which I ask you to acquiesce in with the directors. For years past it has been our custom to devote a certain sum to what we call our benevolent fund. That was established to help any of our employees who fell out from old age or ill health or other causes that might arise, and that fund has accumulated to the extent of between £3,000 and £4,000. It is invested entirely outside the business in the hands of trustees, and we felt that it was not absolutely necessary to add anything to it this year. But we do ask you to sanction a grant of £500 to the building fund of Bartholomew's Hospital. Of course we know that we are quite within our rights in granting this money without asking your consent, but it is much nicer that you should consent to it, and I think there are very good reasons why you should do so. A great number of our employees for years past have been using the hospital as in-patients and out-patients. One of our most valued servants was crushed in the lift, and but for the proximity of the hospital could not possibly have survived. The accident was of a frightful character, but I am thankful to say that he is back amongst us again and doing his work as well as ever. Numbers of cases amongst our employees are continually going into the hospital. You know there has been a great deal said of late as to the hospital not being wanted on its present site. Well, your directors have lived the greater portion of their working lives by the very gates of the hospital. Day by day we see the streams of poor people who are going to that hospital to get relief. From 800 to 1,000 patients go there every day, and we feel that it is a duty on our part to assist the hospital, and I hope you will back me up in that opinion. Well, I take it that you are quite satisfied that the statement in the report shall pass, and I thank you very much. It was stated not long since by a very exalted personage in this City that now all the old firms were being turned into companies it was no longer possible to get subscriptions from the same source. Well, I differ from that statement. I think if the case is fairly and properly put before directors and shareholders they will see it is their duty to continue the good work that was formerly carried on by the private firms. I feel confident that they will answer the appeal if it is properly put to them, and I am very pleased indeed to think that your company should be the first to take the lead in the matter. I am very much obliged to you for your consent.

